# Individual and organizational features of a favorable work environment in nursing homes: a cross-sectional study

**DOI:** 10.1186/s12913-022-08608-9

**Published:** 2022-10-10

**Authors:** Thomas Potrebny, Jannicke Igland, Birgitte Espehaug, Donna Ciliska, Birgitte Graverholt

**Affiliations:** 1 Faculty of Health and Social Sciences, Western Norway University of Applied Sciences, Bergen, Norway; 2 Department of Global Public Health and Primary Care, University of Bergen, Bergen, Norway; 3 Faculty of Health Sciences, McMaster University, Hamilton, Canada

**Keywords:** Organizational context, Work environment, Alberta context tool, Nursing home, Healthcare.

## Abstract

**Background:**

The organizational context in healthcare (i.e., the work environment) is associated with patient outcomes and job satisfaction. Long-term care is often considered to be a challenging work environment, characterized by high job demands, low job control, a fast work pace and job dissatisfaction, which may affect patient care and increase staff turnover.This study aims to investigate the organizational context in nursing homes and the features of favorable or less favorable work environments.

**Methods:**

This study is a cross-sectional study of registered nurses and licensed practical nurses in Bergen, Norway (n = 1014). The K-means clustering algorithm was used to differentiate between favorable and less favorable work environments, based on the Alberta Context Tool. Multilevel logistic regression analysis was used to investigate the associations between individual sociodemographic factors, nursing home factors and the probability of experiencing a favorable work environment.

**Results:**

45% of the sample (n = 453) experienced working in a favorable work environment. Contextual features (especially a supportive work culture, more evaluation mechanisms and greater organizational slack resources) and individual features (having a native language other than Norwegian, working day shifts, working full time and belonging to a younger age group) significantly increased the likelihood of experiencing a favorable work environment.

**Conclusion:**

The work environment in nursing homes is composed of modifiable contextual features. Action in relation to less favorable features and their associated factors should be a priority for nursing home management. This survey indicates that specific steps can be taken to reduce the reliance on part-time workers and to promote the work environment among staff working the night shift.

**Supplementary Information:**

The online version contains supplementary material available at 10.1186/s12913-022-08608-9.

## Background

The organizational context in healthcare broadly refers to the setting or the environment in which people receive or give healthcare, or more simply the sum of forces at work that create the work environment [1]. In healthcare, a positive organizational context is known to promote a wide range of patient outcomes, such as reduced mortality rates, fewer hospital-acquired infections and improved wellbeing [2, 3].

The nursing home setting is different from other healthcare settings as residents are mainly older, dependent adults, often frail with complex care needs towards the end of their lives. Research into nursing homes suggests that a more favorable organizational context can lead to better person-centered care, a higher quality of care, lower rates of drug use and less need to use restraints [4–7]. In addition, previous studies have found that nursing home facilities with more favorable organizational context, had lower rates of urinary tract infections [8, 9] and catheter use [9] among older adults. In addition, staff in favorable nursing home facilities generally reported greater job satisfaction [3], used best practice guidelines more often [9] and provided better treatment in relation to challenging behaviors related to dementia, combined with a more appropriate distribution of antipsychotic medication among older adults [8]. Differences in clinical outcomes and best practice use from an organizational perspective illustrate the importance of considering the influences on the work environment at unit or facility level [10].

In the Norwegian setting, nursing homes provide long-term care for around 12% of all older adults over the age of 80 [11]. The average length of stay in nursing homes is two years [12, 13]. Most residents have comprehensive care needs and over 80% of the residents in Norwegian nursing homes suffer from dementia [14]. Working with bedridden patients, with complex care needs, has been associated with more challenges in nurses’ work environment. Indeed, a study of Norwegian licensed practical nurses (LPNs) showed that the work environment in Norwegian nursing homes was characterized by high job demands and low job control, a fast work pace and frequent exposure to threats and violence [13]. Moreover, such a challenging work environment could affect the quality of care [2, 15]. In addition to affecting the quality of care, organizational work environment factors can have greater societal consequences, as these are associated with turnover intention [16] and the turnover among registered nurses (RNs) and LPNs, working in long-term care, is particularly high in Norway [17]. The high turnover in long-term care is concerning, as health workforce projections indicate that a workforce shortage in long-term care could emerge in Norway by 2035, due to a growing demand for care as a result of an aging population, combined with retirement, a high turnover of nurses and an increase in the drop-out rates of nursing students [17]. Measures intended to improve the working conditions of nurses, so as to increase retention rates in Norway, is recommended [17].

Despite the known challenges in the work environment of RNs and LPNs involved in long-term care, no known studies have investigated the features of a favorable organizational context in Norwegian nursing homes. This study aims to investigate the organizational context of nursing homes and the features of favorable or less favorable work environments.

## Methods

### Study design and participants

This cross-sectional survey was carried out in the municipality of Bergen, the second largest municipality in Norway, with a population of 415,000 inhabitants. We invited all 34 nursing homes in the municipality to participate (census sampling), of which 28 facilities accepted the invitation. Upon participation in the study, one contact person in each facility was appointed to assist with the recruitment of RNs and LPNs. Participants were eligible to participate if they were an [1] RN or LPN and held a position of at least 25% of full-time employment, [2] had worked in the nursing home for at least three months and [3] were able to read and write in Norwegian. The number of eligible nurses in the nursing homes was provided by the nursing home administration (n = 1814). The survey was answered individually. Active consent was given prior to participation in the study.

### Setting

This study was undertaken as a part of the integrated knowledge translation project, IMPlementation and Action for Knowledge Translation (IMPAKT), with the aim of exploring and facilitating the “knowledge-to-action gap” in nursing homes [18].

In the Norwegian setting, the municipalities are responsible for primary care, including nursing home care. Nursing homes in Norway are characterized by the highest density of nurses in Europe [17]. Around 10% of nursing homes are private, non-profit facilities that collaborate with and are funded by the municipalities. Long-term care is provided by RNs, LPNs and nurses’ aides, under the supervision of physicians.

### Measurement

The Alberta Context Tool (ACT) is a valid measure of organizational context in the healthcare setting, which captures healthcare workers’ perceptions of organizational context. These perceptions can be aggregated to provide facility-level estimates of context [1]. The ACT questionnaire contains 56 questions that consist of 10 modifiable concepts relating to organizational context and work environment indicators. The ACT concepts are: [1] leadership (six items), [2] culture (six items), [3] evaluation (six items), [4] formal interactions (five items), [5] informal interactions (seven items), [6] social capital (six items), [7] structural and electronic resources (11 items), [8] organization slack—time (four items), [9] organization slack— staff (two items) and [10] organization slack - space (three items). All items were derived individually, and most were scored on a five-point Likert scale (from strongly disagree to strongly agree) while three concepts (formal interactions, informal interactions and electronic resources) were scored on a frequency scale (from never to almost always). Mean scores were obtained from the Likert scale items, with scores ranging from 1 to 5. Frequency scores were derived by recoding as 0 (never and rarely), 0.5 (occasionally) and 1 (frequently and almost always) and were calculated as the sum of the recoded items. The recoding was conducted in accordance with the ACT manual [1].

The reliability and validity of the ACT instrument has been confirmed in a variety of languages and healthcare settings [19–23], including Norwegian [24].

### Statistical methods

The data were analyzed using the R statistical software and the packages, “stats” [25] and “lme4” [26].

K-means clustering [27] is a commonly used, unsupervised machine learning algorithm for partitioning a given data set into a set of groups, where k represents the number of pre-specified groups in which different clusters are as (dis)similar as possible. Previous studies on the ACT recommend using a k-means algorithm, instead of the mean score, to partition the data into two dissimilar and nonoverlapping groups, in order to facilitate complex organizational structures in a more accessible way, which can be interpreted as favorable and less favorable work environments [8, 9]. We used the k-means clustering algorithm on both individual and nursing home aggregate scores, based on the standardized scores of the 10 ACT concepts. This process essentially allows for the dichotomization of the sample into groups with favorable [1] and less favorable work environments, (0) based on cluster differences [9]. The individual level clusters represent favorable or less favorable work environments and are used for primary statistical analysis, whereas the nursing home clusters were used to describe and examine nursing home level differences. Cohens d was used to quantify the standardized mean score difference in ACT concepts between favorable and less favorable work environments, with d = 0.2 representing a small difference, d = 0.5 representing a moderate difference and d = 0.8 representing a large difference [28].

For the regression analyses, we investigated the features of favorable and less favorable work environments using univariate and multilevel logistic regression analyses, with a random intercept for nursing homes (as respondents are nested within nursing homes). Relevant explanatory or control variables were included if they significantly improved the model fit, based on the Akaike information criterion (AIC), such as gender, type of primary shift worked (day, evening or night), native language (Norwegian or other), educational background, age (centered at 20–24 with five-year intervals), nursing home size (number of beds) and ownership/operator model (public or private).

There was a high correlation between the indicators: [1] years since completion of education, age and years worked at the current nursing home and [2] education level and current professional position. In order to avoid potential multicollinearity, age and education only will be considered for inclusion in the logistic regression analyses. In addition, very few people in the sample had a master’s degree (n = 26), therefore, education was recategorized into high school or higher education in the regression analyses.

Intraclass correlation (ICC), used in this paper, indicates the amount of variance in individual nurse scores that can be explained at nursing home level. An ICC value greater than 0.05 indicates that there is meaningful variation at the group level that warrants further investigation [29].

### Ethical considerations

This research has been performed in accordance with Norwegian ethical guidelines and regulations and approved by NSD - the Norwegian Centre for Research Data (reference number 49,918).

## Results

### Sample characteristics

Among the 28 nursing homes participating in this study, there were a total of 1814 eligible participants of which 1014 RNs and LPNs participated (56%). The response rate ranged from 47 to 76% between facilities. Of the 28 nursing homes, 24 (85.7%) were publicly funded, while the remaining four were private, non-profit organizations. The average size of the nursing homes, measured by the number of beds, was 67.2 (SD = 33.0) for public nursing homes and 76.3 (SD = 65.9) for private, non-profit nursing homes.

93% (n = 932) of the sample were females. The majority were LPNs (60.7%) while the remainder were RNs (39.3%). The majority of nurses worked full-time and primarily worked daytime shifts. The vast majority worked in public nursing homes (Table 1).

**Table 1:** Sample characteristics

Total sample size	1014
**Sex, n (%)**	
Male	73 (7.3)
Female	932 (92.7)
**Age group, n (%)**	
20–39	370 (36.7)
40–59	498 (49.5)
60+	139 (13.7)
**Position, n (%)**	
RN	394 (39.3)
LPN	609 (60.7)
**Completed education, n (%)**	
High school	542 (53.8)
Higher education, bachelor’s degree	439 (43.6)
Higher education, master’s degree	26 (2.6)
**Years since completed education, mean (SD)**	15.1 (11.3)
**Years worked at the current nursing home, mean (SD)**	9.63 (7.9)
**Employment status, n (%)**	
Full time	533 (53.6)
Part-time	461 (46.4)
**Type of primary shift, n (%)**	
Daytime	761 (75.9)
Evening	136 (13.6)
Night	105 (10.5)
**Native language, n (%)**	
Norwegian	747 (74)
Other	263 (26)
**Ownership/operator model, n (%)**	
Public	885 (87.3)
Private (non-profit)	129 (12.7)

### Organizational context and the work environment in norwegian nursing homes

Based on our sample of nurses, the ACT concept’s mean scores from highest to lowest were as follows: social capital (M = 4.02, SD = 0.53), culture (M = 3.96, SD = 0.75), leadership (M = 3.75, SD = 0.77), evaluation (M = 3.41, SD = 0.75), space (M = 3.13, SD = 1.10), staff (M = 2.63, SD = 0.99), informal interactions (M = 2.54, SD = 2.13), structural resources (M = 2.20, SD = 2.25), time (M = 2.20, SD = 0.78) and formal interactions (M = 0.40, SD = 0.74). Based on the k-means clustering assignment of favorable and less favorable work environments, 45% of the sample (n = 453) reported a favorable work environment. (As a sensitivity test, note that these two clusters have a strong correlation with a cut-off at the mean overall ACT score (r = .82, p < .001). All 10 concept scores of the ACT were significantly higher among those in the favorable work environment cluster and the largest differences between favorable and less favorable clusters were found within the organizational, contextual features of *culture* (d = 1.3), *organizational slack- time* (d = 1.2) and *evaluation* (d = 1.1).

K-means clustering analysis on data aggregated at the nursing home level indicates that 15 out of 28 nursing home facilities (54%) had a favorable work environment context, all of which had significantly higher scores on all the 10 ACT concepts, compared to the less favorable cluster. The largest standardized differences between favorable and less favorable nursing homes related to *organizational slack - staff* (d = 0.4), *culture* (d = 0.4) and *organizational slack - space* (d = 0.4).

In summary, where the participating nursing home facilities diverge in terms of being coined as favorable or less favorable work environments, largely depends on their scores in relation to the modifiable, organizational features: culture (the balance between best practice and productivity), organizational slack resources (adequate staffing, physical space and sufficient time to deliver quality care) and evaluation mechanisms.

### Work environmental influences in norwegian nursing homes

Based on the results of the multilevel logistic regression accounting for nursing home differences, individual factors, such as having a native language other than Norwegian, primarily working day shifts, working full time and being younger, significantly increased the likelihood of nurses experiencing a more favorable work environment. Conversely, nurses who mostly worked the night shift were 2.2 times more likely to experience a less favorable work environment, compared to daytime workers. Nurses with a native language other than Norwegian were 1.9 times more likely to experience a favorable work environment than Norwegian speakers. Compared to nurses employed part-time, those who were employed full-time were 1.4 times more likely to experience a favorable work environment. In addition, as nurses get older (and have worked longer in the organization) the odds of experiencing a favorable work environment decreased.

Based on the ICC coefficient, 7% of the variance in individual work environment scores could be explained by nursing home affiliation.

There was no significant difference in the work environment scores based on gender or education level in our sample, when adjusting for other covariates (Table 2).

**Table 2:** Logistic regression results of predictors related to a favorable work environment

	Univariate model			Multivariate multilevel model		
***Predictors***	***Odds Ratios***	***CI***	***p***	***Odds Ratios***	***CI***	***p***
Gender (female)	0.61	0.38–0.99	**0.045**	0.62	0.36–1.06	0.083
Primary shift: evening	0.83	0.57–1.19	0.310	0.83	0.55–1.25	0.369
Primary shift: night	0.47	0.30–0.72	**0.001**	0.46	0.28–0.77	**0.003**
Native language (other than Norwegian)	2.16	1.62–2.88	**< 0.001**	1.92	1.40–2.63	**< 0.001**
Age	0.90	0.86–0.95	**< 0.001**	0.93	0.88–0.99	**0.016**
Employment status (Full-time)	1.81	1.41–2.34	**< 0.001**	1.40	1.05–1.88	**0.024**
Education level (higher edu.)	1.62	1.26–2.08	**< 0.001**	1.26	0.95–1.67	0.105
**Random Effects**						
σ^2^	3.29					
τ_00_	0.23 _Nursing home_					
ICC	0.07					
N	28 _Nursing home_					

## Discussion

Our study aimed to investigate the organizational context in nursing homes and the features of favorable or less favorable work environments among RNs and LPNs working in nursing homes in Norway. Based on our survey of 1014 respondents (56% of the total eligible RN and LPN population) from 28 of a total of 34 nursing home facilities, we found that the work environment varies to a large extent depending on the nursing home. Around half of the facilities were characterized as having a favorable organizational context and almost half of the RNs and LPNs working within these experienced a favorable work environment. In terms of modifiable aspects of the work environment, the most distinguishable differences between favorable and less favorable work environments were having a *positive work culture* (balance between best practice and productivity), *greater organizational slack* (adequate staffing, physical space and sufficient time to deliver quality care) and *more evaluation mechanisms* (feedback mechanisms). On an individual level, having a native language other than Norwegian, working day shifts, working full time and being within a younger age group, significantly increased the likelihood of nurses experiencing a more favorable work environment across nursing homes.

### Shift work and the work environment

Shift work, i.e., working outside the typical daytime work schedule, is known to have a detrimental effect on health, sleep and job satisfaction among nurses [30]. Health problems related to shift work are especially prevalent in a healthcare setting in which patients require around-the-clock care [30]. Our study showed that working night shifts was one of the strongest predictors of experiencing a less favorable work environment. A qualitative study among healthcare workers in Australian hospitals indicated that the work environment experienced by night staff was less favorable by comparison with other shift types, in line with the findings of the current study. The study suggests that night workers may feel disconnected from the organization as a whole and are often less involved in care or organizational decisions. Moreover, they experience minimal support from their non-night working colleagues [31]. However, a positive organizational context may mitigate some of the negative consequences of working the night shift (or evening shifts) to a certain extent [32, 33]. There is evidence to suggest that employees working night shifts experience more positive outcomes when they are allowed input, choice and flexibility in their work schedules [34–37]. In addition, Zhang et al. [33] found that sleep quality and duration improved among shift workers (and especially for night-shift workers) in accordance with each beneficial work environment feature introduced.

Our study highlights that modifying the features of the work environment, such as the work culture, may increase connectedness to the overall work environment among RNs and LPNs in long-term care.

### Working full-time and the work environment

There is a shortage of qualified nurses in long-term care and this shortage is only expected to worsen as the population gets older [17]. Improving the work environment in order to attract nurses to work in long-term care is one measure that should be prioritized, according to the OECD. Improving the work environment through organizational slack has the potential to reduce turnover and increase the recruitment of healthcare workers. This study found that RNs and LPNs, who work full-time, have a greater likelihood of experiencing a favorable work environment, in line with other studies [38]. A study on Spanish nurses indicated that part-time workers generally had lower levels of commitment and engagement in relation to their jobs, compared to their full-time colleagues. Part-time nursing staff also reported lower levels of job resources, such as autonomy and self-development opportunities [38, 39]. A review of the literature also suggested that improved RN staffing was associated with fewer adverse patient outcomes in nursing [40]. Therefore, nursing home management should consider offering full-time positions to the care workers who are working part time involuntarily or should introduce flexibility in work schedules for those who prefer to work part time. This could facilitate higher organizational commitment, particularly in units and facilities where the work environment has been reported as being less favorable.

### Non-native born workers

One surprising finding in this study is that RNs and LPNs, who can read and write Norwegian, despite this not being their primary language, were more likely to report a better work environment compared to their native-born colleagues. The country of origin of the 160 respondents in our study, who were not born in Norway, is unknown. Due to this uncertainty, we should be cautious with regard to the interpretation of these findings. However, it is likely that many of them were born in other Nordic or EU countries, and there might be a “healthy migrant effect” in cases where the non-native speakers are highly motivated to work, compared with the more general population. Another Norwegian study also found that non-native speaking healthcare workers were more satisfied with their job and experienced lower levels of stress compared to their colleagues [41]. The authors speculate that culture, traditions and attitudes could feasibly explain this difference or that experience of other healthcare systems with limited employment security could lead to a greater appreciation of the benefits of having a job [41].

### Organizational features and the work environment

In good work environments nurses are adequately staffed, have adequate resources, supportive managers, strong nursing foundations underlying care, productive relationships with colleagues, input into organizational affairs, and opportunities for advancement [42]. This is in line with our study, where nurses who perceive the work environment as being favorable report experiencing a better work culture, more organizational slack resources, and more feedback on team performance in their workplace.

A systematic review of the evidence suggests a clear link between positive organizational features and workplace culture and improvements in a wide range of patient outcomes such as reduced mortality rates, falls and hospital acquired infections [2]. In addition, nurses in nursing home teams that routinely monitor their performance and receive more feedback from colleagues have higher job satisfaction [43], in line with this study. Thus, teamwork indicators, like feedback from colleagues, may be a key mechanism for staff retention and job satisfaction in the long term [43, 44].

Organizational slack is the cushion of resources (i.e., having enough staff, space and time) which allows care units to deliver high quality care [1]. However, nurses working in nursing home care are often unable to provide necessary care due to insufficient time or resources, which is a stated patient safety concern [15]. Other studies have found that job satisfaction increase substantially when staffing- and resources in nursing homes are adequate [43]. At the organizational level this study found that having more organizational slack resources in general was associated with a more favorable work environment among nurses, in line with previous research. In addition, we found that working full-time was associated with a more favorable work environment at the individual level. It may therefore be fruitful for administrators to consider offering staff who work involuntary part-time in their organization, a full-time position to ensure appropriate staffing levels with qualified staff - when improving the work environment is a stated goal.

### Implications

A key implication of this study is that efforts made to promote positive work environments may have substantial benefits for RNs and LPNs working in long-term care. Interventions that aim to improve specific modifiable organizational features, such as work culture, evaluation mechanisms and organizational slack, may have the potential to improve the work environment of healthcare workers and by proxy, improve patient outcomes and reduce turnover [2]. As a first strategy, full-time employment in nursing homes is a feature of a favorable organizational context that may improve the work environment. By committing to having more experienced staff on hand, it is feasible to ensure more time for quality patient care (organizational slack resources). Secondly, the negative effects of shift working on the work environment, especially the night shift, are well known. Therefore, strategies that can increase organizational integration and the work culture of night shift workers, such as being able to provide an input in relation to patient care and increasing collaboration between the evening and day shift is one option. In addition, allowing flexibility in night workers’ work schedules and providing planned rest periods and exercise options could mitigate some of the negative effects associated with working the night shift. Thirdly, an increased focus on feedback mechanisms from the team and peers, in order to improve staff performance is important in ensuring a healthy work environment. Organizational context and work environment are ultimately an organizational and managerial responsibility. Therefore, in order to improve organizational context and the retention of healthcare workers in long-term care, action should be taken at managerial and organizational levels.

### Strengths and limitations

The sample size of 1014 respondents from 28 nursing homes can be regarded as an appropriate and representative sample from a large municipality in Norway. The participants from the 28 nursing homes represent a selection of facilities with a wide range of organizational contexts, including differences in size, staff, ownership and management structure. Therefore, our study sample reflects the true population of healthcare workers in Norway, in terms of gender distribution and ethnicity [45].

Non-native born workers appear more likely to experience a better work environment and it would have been insightful to have investigated this further. However, missing information regarding the country of birth of the 160 participants, whose first language is not Norwegian, limits further discussion relating to these findings. Furthermore, one general limitation concerns the use of cross-sectional data, which does not permit strict causal inference, therefore, the results should be interpreted with caution.

## Conclusion

The organizational context in nursing homes is often characterized by challenging working conditions, which in turn may negatively influence the work environment. A poor work environment for nurses may affect both the quality of care provided for residents, as well as the recruitment and retention of nurses. Thus, promoting a favorable work environment in nursing home care may benefit both the healthcare workers in terms of their working conditions and improve the quality of care provided.

An encouraging finding of this study is that efforts made to address the modifiable features of organizational context may have the potential to improve the work environment for nurses in Norwegian nursing homes. Specific steps can be considered to minimize the number of nurses working part-time involuntarily and those working night shifts, as night shift workers have an increased risk of feeling disconnected from the overall organization and work environment.

## Electronic supplementary material

Below is the link to the electronic supplementary material.

Supplementary Material 1

## Data Availability

The dataset used and analyzed during the current study are available in the supplementary material.

## List of abbreviations

**ACT**: The Alberta Context Tool.
**LPNs**: licensed practical nurses.
**RNs**: registered nurses.
**IMPAKT**: IMPlementation and Action for Knowledge Translation.
